# The “Heat-and-Cold” Strategy: Combining Intercostal Nerve Cryoablation for Analgesia After Uniportal VATS Pleurectomy and Hyperthermic Intrathoracic Chemotherapy

**DOI:** 10.3390/diagnostics16213490

**Published:** 2026-10-25

**Authors:** Dania Nachira, Giovanni Punzo, Giuseppe Calabrese, Stefano Margaritora, Filippo Lococo

**Affiliations:** 1 Department of General Thoracic Surgery, Fondazione Policlinico Universitario A. Gemelli, IRCCS, Università Cattolica del Sacro Cuore, 00135 Rome, Italystefano.margaritora@policlinicogemelli.it (S.M.); filippo.lococo@policlinicogemelli.it (F.L.); 2 Department of Anesthesiology and Intensive Care Medicine, Fondazione Policlinico Universitario “A. Gemelli”, IRCCS, Università Cattolica del Sacro Cuore, 00135 Rome, Italy; giovanni.punzo@policlinicogemelli.it

**Keywords:** thymoma, pleurectomy, HITHOC, ESPB, cryoanalgesia

## Abstract

We report interesting images from a 54-year-old woman with recurrent B2 thymoma associated with myasthenia gravis, treated using a minimally invasive uniportal VATS pleurectomy combined with hyperthermic intrathoracic chemotherapy (HITHOC). Preoperative imaging identified two hypermetabolic pleural lesions. Complete resection was achieved, followed by cisplatin-based HITHOC. Postoperative pain was managed by a multimodal approach combining erector spinae plane block and intercostal nerve cryoablation, resulting in a VAS score of two on the first postoperative day, decreasing to zero subsequently, and with no opioid requirement to control pain. The patient experienced an uneventful recovery and rapid discharge. This case highlights the feasibility and safety, with favourable outcomes in this patient. Larger and comparative series are needed to confirm the potential advantages of combining minimally invasive surgery with advanced analgesic strategies in complex thoracic oncology.

**Figure 1:**
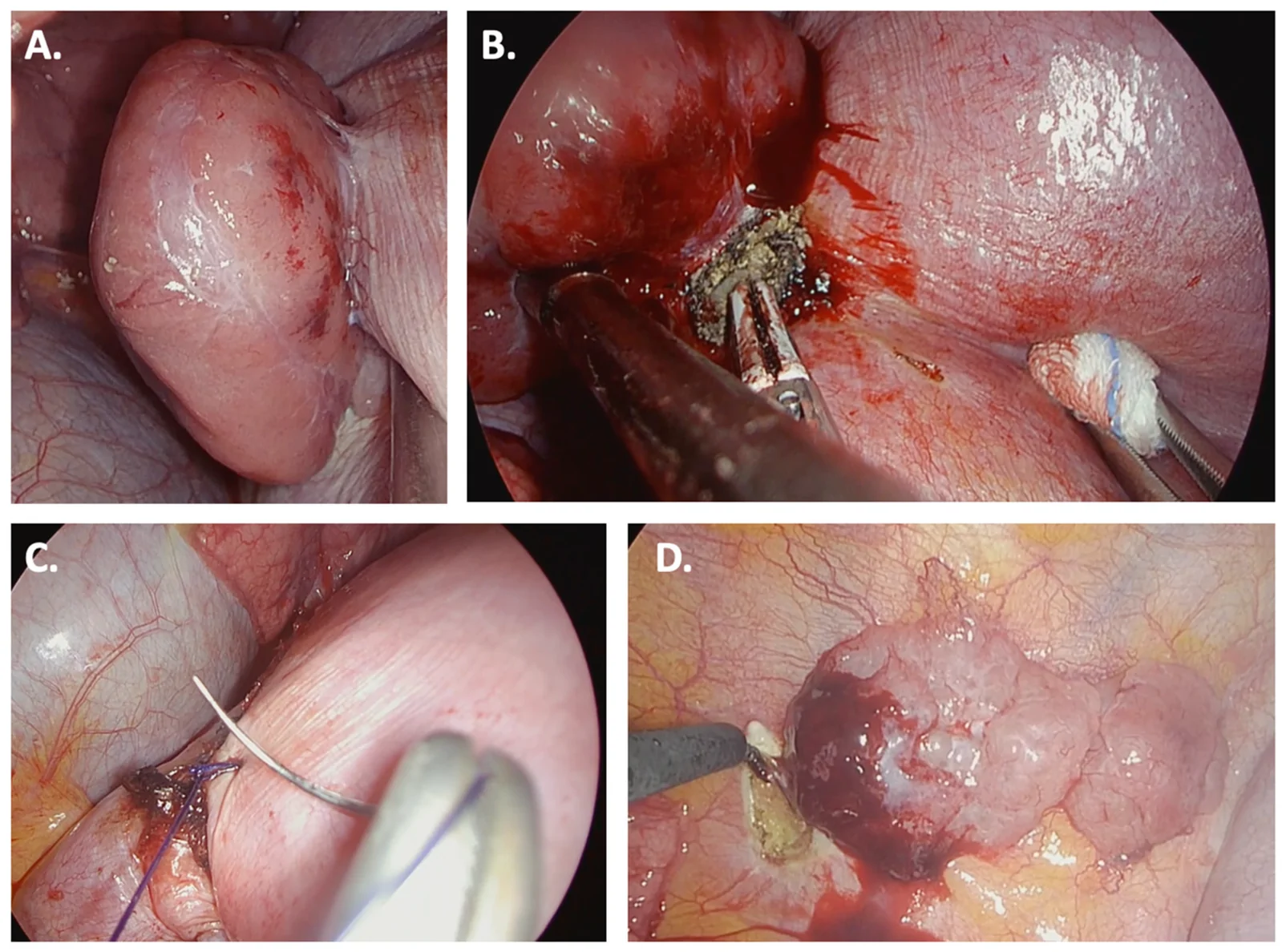
To describe the perioperative application and clinical course associated with combining erector spinae plane block (ESPB) and intercostal nerve cryoablation during Uniportal-VATS pleurectomy and hyperthermic intrathoracic chemotherapy (HITHOC) in a patient with recurrent thymoma and Myasthenia gravis. A 54-year-old woman with a history of B2 thymoma, Masaoka stage IIb, associated with myasthenia gravis, underwent surgical resection in 2012. She subsequently developed pleural recurrence and progressive worsening of myasthenic symptoms requiring plasmapheresis and rituximab. Computed tomography (CT) of the chest demonstrated recurrent pleural disease. 18F-FDG positron emission tomography–CT identified two hypermetabolic pleural lesions: a 5 × 4.5 cm mass overlying the left hemidiaphragm (maximum standardized uptake value [SUVmax] 7.8) and a 2.2 × 5 cm lesion within the costodiaphragmatic recess at the D10–D12 level (SUVmax 6.2). Given her good functional status and preserved renal and hepatic function, the patient was considered suitable for surgical cytoreduction and HITHOC. Preoperative neurological assessment showed well-compensated myasthenia gravis under prednisone 10 mg/day and pyridostigmine 240 mg/day. An additional dose of prolonged-release pyridostigmine 180 mg was administered preoperatively, upon the decision of our neurologists. The planned procedure was a minimally invasive pleurectomy followed by HITHOC. Because extensive pleural dissection and diaphragmatic resection were anticipated, a multimodal regional analgesic strategy combining ultrasound-guided ESPB and intraoperative intercostal nerve cryoablation was planned. The rationale was to provide immediate perioperative analgesia with ESPB while achieving prolonged analgesia through cryoablation, thereby reducing reliance on systemic opioids and avoiding neuraxial analgesia. A uniportal video-assisted thoracoscopic surgery (U-VATS) approach was performed through a 4 cm muscle-sparing incision in the sixth intercostal space along the mid-axillary line. A wound protector, a 30° 10 mm thoracoscope, and long curved double-articulated instruments were used. Thoracoscopic inspection confirmed the two major pleural lesions and revealed additional small suspicious nodules within the costophrenic recess. Pleurectomy included resection of the costophrenic pleura, where the lesions were localized, with macroscopic tumour-free margins, and diaphragmatic pleura, with partial excision of the underlying diaphragmatic muscle (**A**–**D**). The lesions were removed using a no-touch technique (**B**). The diaphragmatic defect was subsequently reinforced with Stratafix™ 2-0 PDS sutures (**C**). No other macroscopic pleural disease was identified. Because of the multifocal pleural involvement and the patient’s preserved renal and hepatic function, HITHOC was subsequently performed with cisplatin at a dose of 150 mg/m^2^ at 42 °C for 60 min, administered in two equal fractions according to institutional protocol. The perfusate volume was 3 L, circulated at a flow rate of 1 L/min using a closed-circuit perfusion system. The inflow temperature was set at 43 °C and the outflow thermometer measured 41 °C, therefore maintaining the intracavitary temperature at a mean of 42 °C. Continuous invasive arterial pressure monitoring and core temperature measurement were maintained throughout HITHOC. Hemodynamic parameters remained stable throughout the perfusion period, with no episodes of significant hypotension, arrhythmia, or temperature-related instability recorded. Renal protection consisted of sodium thiosulfate, 9 g/m^2^ administered 30 min before HITHOC, followed by 9 g/m^2^ from the beginning of perfusion over the subsequent 6 h.

**Figure 2:**
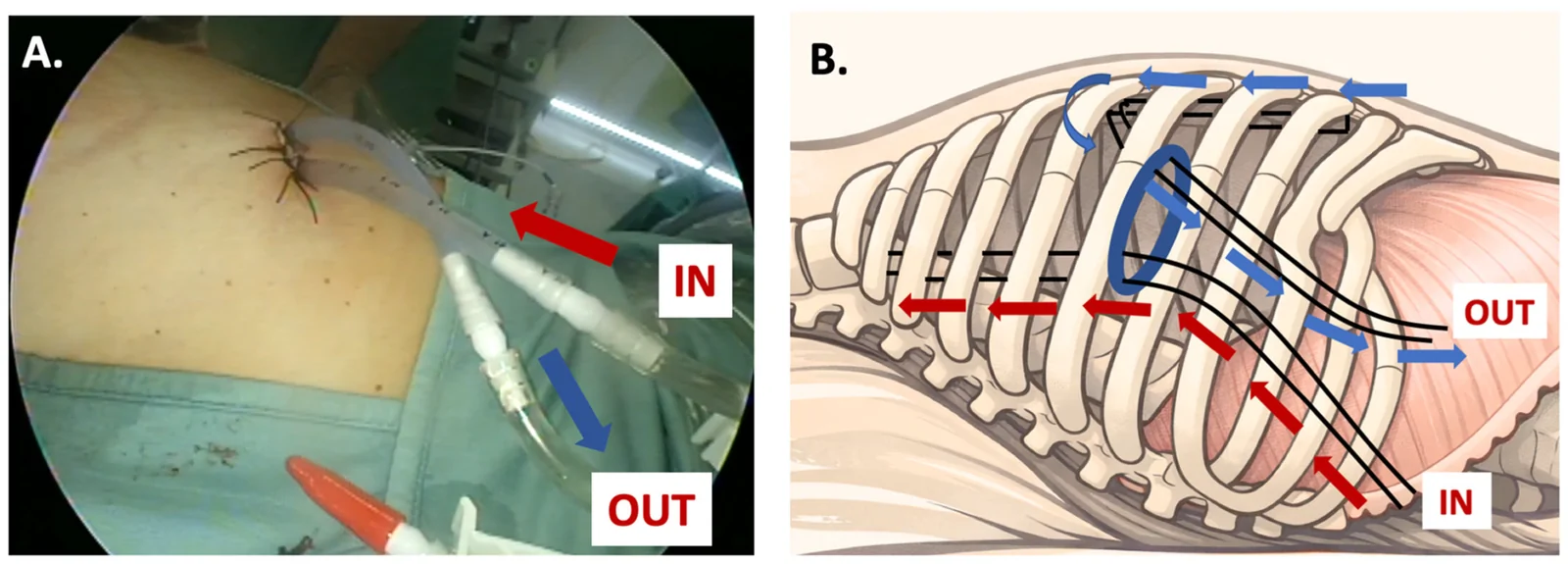
HITHOC perfusion was performed through two 32-Fr chest tubes introduced through the same U-VATS incision (**A**). The tubes were positioned with the inflow and outflow tips separated as widely as possible to optimize distribution of the heated chemotherapy solution throughout the pleural cavity (**B**). At the end of perfusion, the pleural cavity was irrigated with approximately 5 L of saline to remove residual chemotherapy. A 28-Fr chest drain was left in place.

**Figure 3:**
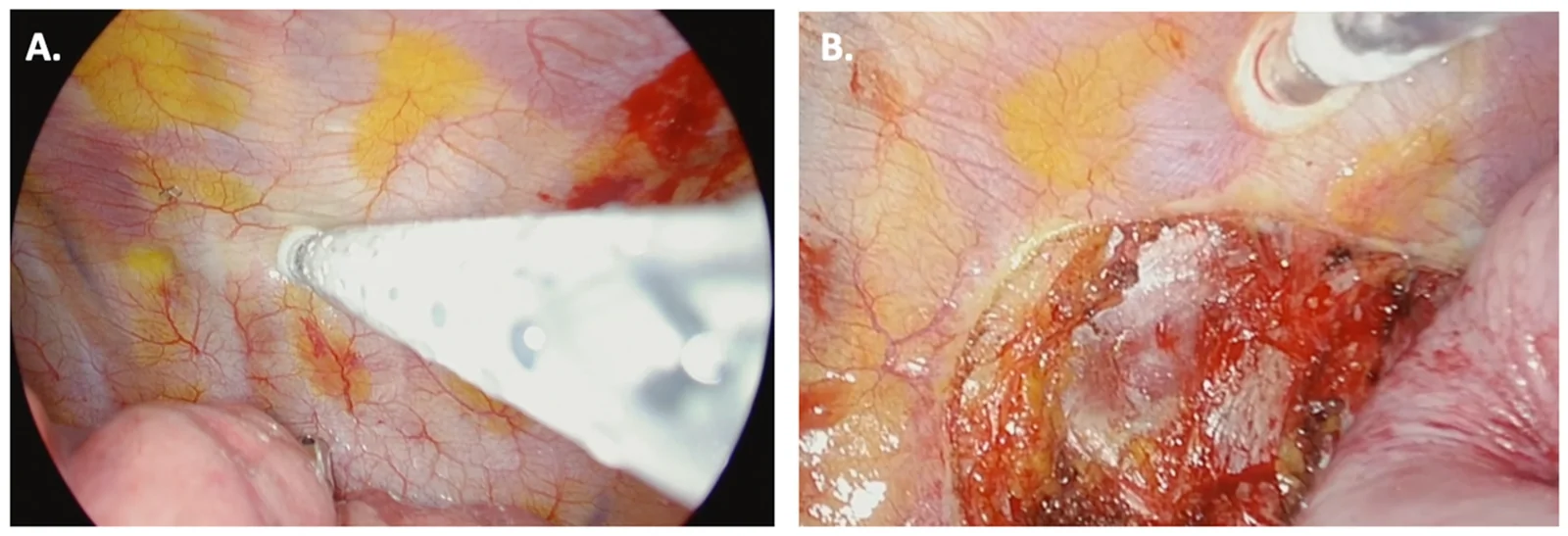
The analgesic strategy was deliberately designed around two regional techniques with complementary temporal profiles: single-shot ESPB for immediate analgesia and intercostal nerve cryoablation for prolonged analgesia, to guarantee a constant and long-lasting pain control. Before induction of general anesthesia, the patient was positioned sitting. Under ultrasound guidance, a total of 30 mL of 0.375% ropivacaine was injected (by a 22-gauge needle), at the T5 level with appropriate separation of the erector spinae muscle from the underlying transverse process. General anesthesia was induced with intravenous propofol (2 mg/kg) and fentanyl (2 μg/kg), followed by rocuronium (0.6 mg/kg) to facilitate orotracheal intubation. A left-sided double-lumen tube was placed under bronchoscopic guidance, and mechanical ventilation was started to maintain end-tidal CO_2_ (EtCO_2_) at 35–45 mmHg. Anesthesia was maintained with sevoflurane (1.5–2.5%, bispectral index-guided) and an oxygen–air mixture. A total dose of 250 mcg of fentanest was administered during surgery. Train-of-four monitoring was maintained throughout the procedure. After completion of pleurectomy, but before HITHOC, attention was directed to the intercostal nerves. Under thoracoscopic visualization and with selective lung isolation, a CryoICE cryoSPHERE cryoablation probe (AtriCure, Mason, OH, USA) was introduced through the U-VATS access and applied directly to the intercostal nerves in the paravertebral region, approximately 10 cm from the costovertebral joint (**A**). The fourth through eighth intercostal nerves were targeted. The T4–T8 range was judged to encompass the primary nociceptive input from both the thoracoscopic incision (VI intercostal space) and the resected pleural surface. Each nerve underwent a single 2 min cryoablation cycle at −70 °C (including a continuous freeze + thaw period). The positioning of the probe and the adequate probe–nerve contact were directly visualized thoracoscopically by the operating surgeon, allowing controlled application to the intended intercostal nerves while avoiding adjacent structures (**B**). The whole cryoablation procedure lasted 10 min. The timing of cryoablation immediately after pleurectomy was intentional. Intercostal nerve cryoablation induces a reversible axonotmesis-like injury, interrupting nociceptive transmission, due to a Wallerian degeneration of axonal fibres while preserving the surrounding connective tissue framework (a pattern consistent with Sunderland grade II injury), which accounts for both its analgesic efficacy and the expectation of spontaneous neural recovery [1]. Its analgesic effect is therefore expected to persist considerably longer than that of a single-shot local anesthetic block. However, because the clinical analgesic benefit of cryoablation may not be maximal during the first postoperative hours, the ESPB was used to provide immediate coverage during this transition period, providing broad unilateral thoracic analgesia while avoiding neuraxial instrumentation. Systemic multimodal analgesia consisted of ketorolac 30 mg and paracetamol 1000 mg. No epidural catheter or other neuraxial analgesic technique was used. The procedure and anesthetic course were uncomplicated. Following completion of surgery, sugammadex was used for reversal of neuromuscular blockade. This approach was selected to ensure complete and predictable reversal of neuromuscular blockade, minimizing the risk of residual paralysis—a concern of particular relevance in myasthenic patients. Therefore, the patient was extubated uneventfully and transferred to the post-anesthesia care unit (PACU), where continuous pulse oximetry and respiratory rate monitoring were maintained. No episodes of respiratory depression or oxygen desaturation were recorded. Pain was assessed using a visual analogue scale (VAS; 0–10) by the same surgeon throughout the postoperative period. In the PACU on postoperative day 0 (at 3, 6 and 12 h after surgery), the maximum reported VAS score was 4/10. Intravenous paracetamol 1000 mg every 8 h was administered as part of the multimodal regimen and discontinued once pain was consistently ≤3/10. Intravenous ketorolac (30 mg maximum two times per day) was scheduled as rescue therapy but never administered. On postoperative day 1 (24 h after surgery), the maximum dynamic and static VAS score was 2/10, subsequently decreasing to 0/10 since the second postoperative day. No clinically relevant breakthrough pain requiring rescue opioid administration was recorded. The patient was mobilized early and had an otherwise uncomplicated postoperative course. In particular, no early cisplatin-related toxicity was observed during the hospital stay, including no significant renal function deterioration. Myasthenia gravis was well controlled in the postoperative period with prednisone 10 mg/day and pyridostigmine 240 mg/day. The patient was discharged on postoperative day 5 with a VAS score of 0/10 and without analgesic requirements. At the 2-week follow-up visit, she remained pain free and required no additional analgesic intervention. Importantly, during more than 6 months of follow-up, there was no clinical evidence of persistent neuropathic pain or chronic post-thoracotomy pain, and the patient was disease-free. Histopathological examination confirmed recurrent B2 thymoma. Pleural dissemination of thymoma represents a challenging therapeutic scenario. Although thymomas are generally indolent, their limited responsiveness to systemic chemoradiotherapy means that complete macroscopic resection remains an important component of treatment when technically feasible. HITHOC has increasingly been incorporated into multimodal treatment strategies for pleural dissemination, including stage IVA thymoma, with encouraging oncological outcomes in selected patients [2,3,4]. HITHOC aimed at controlling locoregional disease, based on the direct cytotoxic effect of heated chemotherapy on residual microscopic pleural deposits following surgical resection [2,3,4] The rationale in this case was supported by the patient’s good functional status, preserved renal and hepatic function, and the technically resectable nature of the pleural recurrence identified on preoperative imaging. These procedures are often performed through an open approach, associated with delayed recovery, potentially severe postoperative pain, and risk of chronic neuralgia. In this case, surgery was performed using a minimally invasive U-VATS approach, offering reduced invasiveness and satisfactory short-term outcomes. The present case is also relevant from an anesthetic perspective because an extensive pleural procedure was performed avoiding thoracic epidural analgesia. The principal feature of the perioperative strategy was the combination of intercostal nerve cryoablation and ESPB, which was selected to address different phases of postoperative nociception. Intercostal nerve cryoablation has emerged as an effective regional analgesic technique in thoracic surgery. By producing controlled temporary interruption of axonal conduction, cryoablation can provide analgesia extending well beyond the duration achievable with conventional single-shot local anesthetic techniques. Furthermore, even if delivered via continuous infusion, ESPB would have provided analgesia for a limited period, insufficient to prevent the potential development of chronic postoperative pain in such an extensive procedure. Previous clinical studies have reported effective postoperative pain control, reduced opioid requirements and a potentially lower incidence of chronic post-thoracotomy pain following intercostal cryoanalgesia [5], as observed in this case. Nevertheless, intercostal nerve cryoablation can carry a risk of temporary neurological sequelae, including intercostal hypoesthesia, dysesthesia, allodynia, and chest wall muscle weakness, resulting from the reversible axonotmesis induced by freezing. These effects are generally expected to resolve within 4 to 12 weeks, although individual variability exists, as axonal regeneration occurs along the preserved connective tissue framework (1). Therefore, patients should be informed of these potential effects preoperatively, and longer follow-up in larger series will be necessary to characterize the true incidence and duration of neurological sequelae associated with this technique in the context of pleurectomy and HITHOC.The temporal profile of cryoanalgesia, however, is particularly relevant when considering its integration into a multimodal analgesic strategy. The maximal analgesic benefit of cryoablation may not coincide with the immediate postoperative period. Consequently, relying exclusively on cryoablation could leave a potential gap in analgesic coverage during the first postoperative hours. In the present case, this limitation was addressed by combining cryoablation with a preoperative ESPB [6]. The ESPB provided an immediate, broad unilateral thoracic sensory block from the moment of emergence from anesthesia. Cryoablation, performed under direct thoracoscopic vision before HITHOC, was intended to provide prolonged interruption of intercostal nociceptive transmission after the initial effect of the ESPB subsided. The two techniques were therefore complementary rather than redundant: ESPB provided immediate analgesia, whereas cryoablation was intended to extend analgesia beyond the expected duration of the local anesthetic block. This temporal complementarity represents a meaningful innovation in complex thoracic procedure and in the context of myasthenia gravis, as safe opioid-sparing strategies remain undescribed in the literature. Furthermore, pleurectomy and diaphragmatic resection can generate substantial somatic and parietal pleural pain, while the minimally invasive access itself still involves intercostal nerve trauma. HITHOC may further contribute to postoperative discomfort through thermal and chemical irritation of the pleural surfaces. Effective control of this nociceptive burden is essential not only for patient comfort but also for respiratory physiotherapy, mobilization and recovery. In this patient, the combination of regional techniques and non-opioid systemic analgesia was associated with very low postoperative pain scores. No rescue opioid therapy was required, and the patient remained pain-free after discharge. The approach may also be particularly attractive in patients in whom opioid minimisation is desirable. In this case, the presence of myasthenia gravis provided an additional rationale for an opioid-sparing strategy, given the potential vulnerability of these patients to respiratory depression and prolonged neuromuscular effects. Avoiding thoracic epidural analgesia also simplified perioperative management in the context of minimally invasive surgery and facilitated early mobilization, while reducing the risk of postoperative respiratory depression. To the best of our knowledge, and based on a search of PubMed and Embase conducted in August 2026 using the following search terms: (“intercostal nerve cryoablation” OR “intercostal cryoanalgesia”) AND (“erector spinae plane block” OR “ESPB”) AND (“VATS” OR “video-assisted thoracoscopic surgery” OR “pleurectomy” OR “HITHOC” OR “hyperthermic intrathoracic chemotherapy” OR “thymoma”), this is the first reported case describing the combination of thoracoscopic intercostal nerve cryoablation and ESPB during U-VATS pleurectomy with HITHOC for recurrent thymoma. We wish to clarify that the proposed novelty does not reside in either technique individually—both have been described separately in thoracic surgery—but in their deliberate combination within this specific surgical and clinical context. In this patient, the combination of intercostal nerve cryoablation and ESPB was feasible and was associated with a favourable postoperative analgesic course, including low pain scores and absence of opioid requirements. Whether this approach is generally safe, superior to alternative analgesic strategies, or broadly effective cannot be established from a single case. Prospective studies with larger patient cohorts are needed to evaluate its reproducibility and clinical impact in complex thoracic procedures.

## Data Availability

The original contributions presented in this study are included in the article. Further inquiries can be directed to the corresponding author.

## Abbreviations

The following abbreviations are used in this manuscript:

HITHOC	Hyperthermic intrathoracic chemotherapy
ESPB	Erector spinae plane block
CT	Computed tomography
SUVmax	Maximum standardized uptake value
U-VATS	Uniportal video-assisted thoracoscopic surgery
PACU	Post-anesthesia care unit
VAS	Visual analogue scale
EtCO_2_	End-Tidal carbon dioxide
